# Westminster Hospital

**Published:** 1832-01

**Authors:** 


### HOSPITAL REPORTS.

### WESTMINSTER HOSPITAL.

*Clinical Lecture by* G. J. Guthrie, F.R.S., *on a Case of Iliac Aneurism, in which he tied, with success, the External Iliac Artery.*

The case of John Hicks, on whom I operated for iliac aneurism thirty days ago, is now so far advanced that I can with propriety draw your attention to all the circumstances attending it; many of which have been very valuable in regard to the information they conveyed to those who have watched the progress of the case, and must be important to all. The man is twenty-eight years old, and of a good constitution, as the result of his treatment has shewn. The aneurism was situated above Poupart's ligament, which was raised by the lower part of the tumor, and by its indentation seemed to divide it into a large and small portion, the latter being below, the former above, occupying, as far as I could judge, a space equal to the lower half of the external iliac artery, and being as large and as prominent as a good-sized orange; the pulsation very strong and distinct. During the month preceding the operation, I had him blooded three times: the pulse remained, however, generally above ninety, and sharp. There was a slight bellows sound at the heart, but I believe the arterial system to have been in other respects sound; the aneurism, as far as he could recollect or judge, having taken place from a strain. The situation and size of the tumor prevented the usual operation from being attempted, and supposing it probable that the greater part of the external iliac, if not the whole, might be unsound, I decided on performing the operation so as, if necessary, to place the ligature on the common trunk of the right iliac arteries. This operation has been done three or four times, but not successfully, and is one requiring great coolness and a correct knowledge of the parts to be divided; that kind of knowledge which cannot be acquired from books, casts, or models, and which dissection alone can give; for unless you are perfectly aware of every part you must meet with, and when you are to meet with it, the probability is you will fail in effecting your object, and kill your patient.

I began by making an incision in the side of the abdomen, extending from about an inch within the ninth rib, and about one inch above the level of the umbilicus, to nearly an inch within the ileum. This incision was six inches long, and the integuments, superficial fascia, and the external oblique tendon, were divided as rapidly as possible, until the fibres of the internal oblique were fairly exposed: these, being of uncertain thickness, were next divided cautiously, until the tendinous expansion of the transversalis was brought into view, going to form the sheath of the ureters. The lower portion of this was muscular, but I divided the tendon immediately above this part very easily, introduced a director upwards and downwards, and cut the transversalis upon it in both directions. The peritoneum was now exposed, covered, however, still by the fascia transversalis, which in places fastened and bound it down so firmly as to require to be divided with greater caution by the knife and director. This obstacle being removed, the peritoneum rose and fell with the intestines on each motion of the muscles of the belly, shewing how carefully the preceding steps of the operation must be done to avoid doing it an injury, which at this part might be fatal. I now separated the peritoneum from the fascia transversalis and transversalis muscle, and endeavoured to pass my hand behind towards the spine, between the ribs and the ileum; but I found there was not quite room for it to pass easily: I therefore enlarged the incision as much downwards as the proximity of the aneurism would permit, and divided the transversalis tendon upwards for about half an inch more. As it was here very firmly passing across, I raised the part to be divided on my two forefingers, and desired an assistant to divide it with a pair of blunt scissors: in doing this, a small fold of peritoneum was caught and divided, to the extent of near a quarter of an inch. It was, however, out of the way, and my hand could now pass with perfect ease, although it required to be done with the greatest caution, raising and separating the peritoneum before it, and passing over the psoas muscle, until my forefinger rested on the common iliac. It was only at this part of the operation that assistance could be given by any one: Mr. White now, however, took charge of the bag of peritoneum containing the intestines, and raised it and them sufficiently to enable me to see the point of my finger resting on the vessel and on the bone beneath. I could now feel, and indeed see, the common iliac and its bifurcation into the internal and external iliacs, and it was as easy to have placed a ligature on any one as on the other of these three arteries, and more particularly on the common trunk. The external iliac appearing, however, to be sound, and of its natural size and appearance, an inch below the bifurcation, I cleared it with a blunt knife, and passed a ligature around it from within outwards, made of two strong threads of dentist's silk, well waxed. I then cut away one end, laid it straight, and allowed the peritoneum and intestines to fall back into their places. If I had a doubt about the soundness of the external iliac, I should have tied the common trunk; but as it was, and as I could with propriety place the ligature an inch below the bifurcation, I did it, and of course gave my patient an additional chance of escaping mortification.

The wound was first brought together by three strong ligatures passed through the integuments, leaving the muscular wall to itself; and as these did not close the wound, four stitches through the skin, of a single thread each, were added in the interstices, which brought the edges of the whole line of incision into contact. The adhesive straps were then applied, and a compress was retained by other straps and a flannel roller. The legs were both raised by a pillow under the knees, the body bent, and the patient inclined towards the affected side.

I must now draw your attention to the appearance of the peritoneum when fairly exposed, and, although at the time I was annoyed at the little snip which was made in it, I am now glad it happened, because I have said, in the description of this operation in my work on the Arteries, that a slight injury to it would not be of any consequence; which opinion was in this instance proved to be correct; and that the difference between the colour and appearance of the intestine and the peritoneum was so well marked as not to be forgotten by any one who saw it. The reddish colour and slight roughness of the external surface of the peritoneum exposed by dissection was well contrasted with the leaden colour of the opposite surface of the same peritoneum covering the intestine.

I performed this operation, naming every part as it was divided or separated, precisely as I should do if I were shewing you an operation on the dead body. I always, as you know, do so as far as time will permit; and if you will take my book with you into the dissecting room, and follow every step of the operation as it directs, taking every measurement with exactitude, you will be able to do it also in a similar manner. If the operation was in itself instructive, the after-treatment is more so: there were three dangers to be obviated; of mortification, of inflammation, and of hemorrhage, on or about the time of the separation of the ligature.

The temperature of the limb previously to the operation, on Saturday the 19th of November, was 102; the pulse ninety-two. On being put to bed, a hot bath was applied to the foot, and two persons were directed to rub the foot, leg, and thigh constantly under the bedclothes, which they did until midnight. At eight P.M. the pulse was 106, the temperature ninety-two. At twelve on Sunday the pulsation of the femoral artery was distinguishable in the middle of the thigh, and he was free from pain in the limb, which I considered to be a most important sign of safety from mortification; for you may lay it down as a general rule, that, when a limb is going to mortify after such an operation, there is always great pain in the heel, calf, and even in the thigh, whilst at the same time there will be a mottled appearance of the skin, which together are the forerunners of an inevitable and generally fatal mortification. In the evening, the pulsation of the posterior tibial artery was just discoverable at the heel; and on the Monday, or forty-eight hours after the operation, I considered the danger of mortification to have passed by, and that of inflammation to have commenced. The pulse rose to 134, was jerking, but neither full nor hard. I had him bled to nine ounces, when the pulse diminished in volume nearly three fourths, and he felt himself relieved from an oppression he could not describe before. The abdomen was a little swelled and tympanitic, but not painful any where on a fair degree of pressure. An enema, with Ol. Ricini, was given, and repeated with Ol. Terebinth., with full effect. In the evening the pulse was 140, and very peculiar, there being seventy strong beats, and seventy short feebler ones following them; the countenance a little anxious. I had him bled, with my finger on the pulse, until twenty-two ounces were drawn, when he became faint, a profuse perspiration came over him, and the limb lost in temperature.

I may say to you now, in regard to the abstraction of blood, that it was very desirable not to produce positive syncope, yet to go sufficiently near it to repress inflammatory action; for, if syncope had taken place, it is possible the limb might have been lost. I was guided by this in the different bleedings which he underwent during the eight subsequent days, until 107 ounces were taken away in all; the blood, until the last, being cupped and buffed, and firm in the coagulum.

He was kept on four ounces of bread and tea only each day, for the first fortnight, and on eight ounces, with a pint of milk, for the second. The loss of blood was not sufficient, even with the starvation, to subdue inflammatory action, and he took colchicum and digitalis also, until the intermission of the pulse, on the 3d of December, warned me to desist from their use.

The ligature came away on the twenty-eighth day. The violence of inflammatory action induced me to suspect a greater degree of inflammation in the coats of the artery than was compatible with safety; and the removal of the ligature gave me great satisfaction, in assuring me of the soundness of the upper portion: the lower one seems to have inflamed, and the tumor, it would appear, partook of it also; for, on the 13th of December, it was evidently softer, and increasing towards the line of incision, into which it discharged itself the next day, being thereby diminished in size. It still continues to discharge itself in a similar manner, but is so much diminished as to be scarcely perceptible, and I have every reason to hope will become consolidated with the surrounding parts.

The man is now taking porter, fish, and beef tea; sleeps well; is free from uneasiness; and, as far as the operation goes, it has perfectly succeeded, and will form, I presume, a precedent likely to be followed in all similar cases.

It is especially worthy of remark, that the peritoneum seems scarcely to have inflamed beyond the part necessary for the cure, and that even this was confined to the outside. It was the general inflammatory disposition which required a decisive treatment, and not the local, which never exceeded its due bounds. I have said, when writing on the operation for placing a ligature on the aorta, that it must be done in a similar manner; and when my finger was resting on the trunk of the iliacs, I felt confident that I could with ease have placed it on the aorta. I laid bare one inch of the vessel: many persons saw it at a distance in the theatre, and, as the vessel does not exceed two inches and a half in length in the person, it is plain that very little more required to be done. I have no doubt but a farther extension of the external incision for an inch upwards would have given quite sufficient space to have tied the aorta with ease; and I attribute the success of the operation on Hicks to the extent of the incision, which allowed of every step of the operation being done with facility.

We witnessed the performance of the above-described operation, and, in justice to Mr. GUTHRIE, we are bound to state, that we never saw more coolness or more manual dexterity evinced than was by him on this very important occasion.—EDITOR.

---

### *Substance of a Clinical Lecture delivered by* Mr. GUTHRIE, *on a Disease of the Testis.*

I HAVE several times drawn your attention to the case of George Nicholson, as being peculiar and deserving of your best attention: he was admitted on September 9th, being twenty-one years of age, and looking very pale and ill; having the scrotum of the right side very much distended and thickened, forming a tumor of an oval shape, and as large as the head of a child of a year old. My notes say, the skin feels thickened, is more vascular than usual, and of a copper colour; the tumor is soft, elastic, and very weighty; the usual sensation of fluctuation is not experienced on tapping it with the fingers, and the light from a candle does not pass through it. The right testicle has been larger than the left, he thinks, for the last six years, but why he knows not. A year ago he had a gonorrhœa. About two months back, after working all day, lifting great weights, he felt pain in his back and groin, and next morning he found his right testicle swelled and painful; for which he had leeches applied, and was kept in bed. At the end of three weeks he was better, and went to Margate, when the complaint returned, and he came to town to me, and was admitted into the hospital.

The history would lead us to say it was a common inflamed testis, but the appearance of the scrotum shewed it was something more: it was distinctly not a swelling containing serum; it was heavier, had a more doughy feel, gave no sign of fluctuation, was not transparent, and the skin was more vascular. I particularly drew your attention to all these circumstances, and desired you to poise the part on your hand, giving it as my opinion that there was an enlarged, if not diseased, testis, with a quantity of matter in the tunica vaginalis, between blood and pus, and of the consistence of suet partly melted. To prove this, I passed a trocar into the tumor, and a small quantity of the contents came through it, being such as I had predicted: I therefore laid open the tunica vaginalis, and evacuated a considerable quantity of this kind of matter, leaving a testis enlarged to the size of a large double fist at the upper part of the wound. You will recollect there were several surgeons present, not of the hospital, and the opinion was general among them that it was useless to attempt preserving the testis; and I have not the least doubt but that, twenty years ago, it would have been removed forthwith. I told you, however, at the time that its size signified nothing; and that, as it was smooth and equable in its feel, and not much differing from that of the part in its natural state, I should give it a trial. A piece of lint was laid over it and between the edges of the incision, some bands passing between, the testis and the tunica vaginalis having previously been broken down, and a poultice applied over all. The tunica vaginalis suppurated freely; the cellular structure between it and the skin did the same, and the matter had to be evacuated; but the result was the gradual diminution of the whole, and at the end of three weeks the safety of the testis and the certainty of recovery became apparent, the man looking in good health to what he was on admission. I have since given him mercury, both internally and externally; and, although the testis is not yet of its proper size, the case may be said to be completed.

I passed the trocar into the swelling to explore its contents, because the patient would not consent to the incision. It is, however, a good practice in most cases, and often removes a doubt: it did so in this instance, and the patient the more readily submitted to the incision. In making this, it is usual to begin at the upper part, and continue it along the face of the swelling: it will be better, however, always to make it in the middle, and continue it to the under part, which prevents a bag being formed by the inferior portion of the tunica vaginalis, capable of retaining any secretions which may take place, and being often, in consequence, a source of much pain and inconvenience in the course of the subsequent treatment. The operation should always be done in this manner when a hydrocele is cured by incision; and the greatest care should be taken to introduce into the bag of the tunica vaginalis small pieces of lint, which may in a similar manner be retained for two or three weeks, giving rise to much more pain than is necessary.

I wish, however, to draw your attention particularly to the enlargement of the testis: this you saw was considered by several gentlemen of standing and ability as a point of importance; I considered it of none. I wish to impress it upon you, in regard to all soft and equable enlargements of the testis which take place with hydrocele. When the testis, in this disease, is felt to be enlarged, it is usual to abstain from the radical operation, and be content with drawing off the fluid, until the disease of the testis shall be cured. It is almost an axiom in surgery, not to proceed with the radical operation by injection, after drawing off the fluid, if the testis be found greatly enlarged. This is, nevertheless, a mistake: a great number of cases have so thoroughly convinced me of this, that I express the axiom differently, and say that the enlargement of the testis renders the operation by injection necessary: nay, further, that if the enlarged testis be a chronic, soft, or even moderately hard, yet equable, enlargement, with very little fluid, or with fluid even in parts of the tumor, the injection of the whole of these parts is the best mean of cure; and if this should fail, as it may do sometimes, an incision through the tunica vaginalis, in the manner I have pointed out, will scarcely ever fail of effecting a cure. I have had a great number of these cases, which I shall some of these days bring together in one view, and have been invariably successful, even when the patients, from previous treatment, have despaired; and I recommend this plan of treatment, therefore, to you on the ground of experience. It is a point in surgery you will not find stated in this way in books, and therefore it is one to which I particularly draw your attention.

